# Impact of South Asian brick kiln emission mitigation strategies on select pollutants and near-term Arctic temperature responses

**DOI:** 10.1088/2515-7620/ac0a66

**Published:** 2021-06-25

**Authors:** Brannon Seay, Anna Adetona, Natasha Sadoff, Marcus C Sarofim, Michael Kolian

**Affiliations:** 1 Battelle Memorial Institute. 505 King Ave, Columbus, OH 43201, United States of America; 2 United States Environmental Protection Agency. Office of Air and Radiation. 1200 Pennsylvania Ave. NW, Washington, DC 20004, United States of America

**Keywords:** brick kiln, Arctic climate, air quality, aerosol emissions, black carbon, short-lived climate forcers, South Asia

## Abstract

The brick kiln industrial sector in South Asia accounts for large amounts of short-lived climate forcer (SLCF) emissions, namely black carbon (BC), organic carbon (OC), and sulfur dioxide (SO_2_; the precursor to atmospheric sulfate [SO_4_]). These SLCFs are air pollutants and have important impacts on both human health and the Arctic, a region currently experiencing more than double the rate of warming relative to the global average. Using previously derived Arctic equilibrium temperature response factors, we estimate the contribution to Arctic temperature impacts from previously reported emissions of BC, OC, and SO_2_ from four prevalent South Asian brick kiln types (Bull’s Trench [BTK], Down Draught [DDK], Vertical Shaft [VSBK], and Zig-zag). Net annual BC (115 gigagrams [Gg]), OC (17 Gg), and SO_2_ (350 Gg) baseline emissions from all four South Asian kiln types resulted in 3.36 milliKelvin (mK) of Arctic surface warming. Given these baseline emissions and Arctic temperature responses, we estimate the current and maximum potential emission and temperature mitigation considering two kiln type conversions. Assuming no change in brick production, baseline emissions have been reduced by 17% when considering current BTK to Zig-zag conversions and have the potential to decrease by 82% given a 100% future conversion rate. This results in a 25% and 119% reduction in Arctic warming, respectively. Replacing DDKs with VSBKs increases baseline SLCF emissions by 28% based on current conversions and has the potential to increase by 131%. This conversion still reduces baseline warming by 31% and 149%, respectively. These results show that brick kiln conversions can have different impacts on local air quality and Arctic climate. When considering brick kiln emissions mitigation options, regional and/or local policy action should consider several factors, including local air quality, worker health and safety, cost, quality of bricks, as well as global climate impacts.

## 1. Introduction

South Asia (India, Pakistan, Bangladesh, and Nepal) is responsible for over 20% of the 1.5 trillion clay bricks produced each year globally [1]. India alone accounts for over 10% of global production [2] and consumes approximately 31 million metric tons of coal annually in the process [3]. Although considered a small industry sector, large amounts of particle and gaseous air pollutants may be emitted by brick production, namely when simple combustion processes are used. Traditional small-scale brick kilns predominate in South Asia and are characterized as a poor combustion technology with minimal to no pollution control [1], which results in higher black carbon (BC), particulate matter (PM), and carbon monoxide (CO) emissions and lower sulfur content. Stack emissions of BC from brick kilns in India have been measured to be upwards of 100 000 metric tons per year [3], and account for 80% of all BC emissions from brick kilns in South Asia [4].

Some work on estimating emissions from various brick kiln technologies in India has already been reported and suggests that substitution of cleaner kiln types will contribute towards less air pollution (reducing CO and PM emissions by 60 to 70%) and better production yield for the brick industry in India [1, 4]. For instance, traditional kilns such as clamps, downdraft (DDK), and fixed chimney bull’s trench kilns (BTKs) produce greater amounts of emissions compared to vertical shaft brick kilns (VSBK) and Zig-zag kiln technologies [1, 4]. Illustrations and details on the operation of these kilns are provided in Supplemental figure S1 of Weyant *et al* (2014) [1]. Coal is the primary fuel used in each of these kiln types except DDKs, which are biomass-fueled [1, 2]. Brick-producing facilities in South Asia primarily use BTKs (~70%) and clamp kilns (~25%), both of which are poor combustors [1, 5]. These inefficient and fuel-demanding kilns produce significant amounts of greenhouse gases (GHGs) and BC emissions [6]. In addition, manual labor is required to operate these kilns and workers are at high risk of harmful exposures to respirable PM, thermal stress, and injuries [5].

South Asian brick kiln emissions present several public health hazards [7–10] to on-site workers and people living nearby. A recent study [11] reports that mitigating BC and co-emitted pollutants can significantly decrease premature mortality. Given building construction is expected to grow by 6.6% annually through 2030 [2, 4], driven by 10% annual population growth [12, 13], and fired clay brick production is expected to continue dominating building material market shares [4], the negative air quality and public health impacts will be exacerbated in the future. Unless cleaner technologies for brick production are employed, the negative impacts of brick kiln emissions in India will continue to be a challenge.

Several brick kiln air pollutants also impact the Earth’s climate. BC is a carbonaceous aerosol formed primarily in flames and strongly absorbs visible light. Organic carbon (OC) is a reflective carbonaceous aerosol often co-emitted with BC. Sulfate is also a reflective aerosol, created by atmospheric oxidation of SO_2_ which is produced from combustion of high-sulfur fuels (generally coal). The net climate effects of aerosols from a given combustion source will depend on the relative quantities of warming (BC) and cooling (SO_2_ and OC) pollutants produced. Many brick kilns have a high ratio of BC:(SO_2_ + OC) emissions [1, 14]. Therefore, reducing emissions from these kilns has the potential to reduce the near-term rate of Arctic warming [14, 15], a region where near-surface temperatures have increased by as much as three times that of the global average [15–18]. BC, OC, and SO_2_ are considered short-lived climate forcers (SLCFs), given their atmospheric lifetimes are much shorter than CO_2_. However, since the term SLCF often implies the inclusion of methane (CH_4_), which is not analyzed in this study, herein we use the term climate-active aerosols (CAAs) to describe BC, OC, and SO_2_. Research on brick kiln emissions from South Asia indicates that radiative forcing (integrated over 100 years) from one year of brick production emissions is estimated to be about 1 terawatt (TW) per year (yr) for short-lived pollutants and 3 TW yr^−1^ for longer-lived pollutants [1]. These radiative effects are comparable to the approximate 5 TW yr^−1^ of forcing (integrated over 100 years) resulting from one year of U.S. passenger vehicle emissions [1].

Though brick kilns have been assessed regarding worker health and safety, brick quality, cost, global radiative forcing, and air quality impacts, to date no research has reported the impacts of brick kiln emissions on Arctic temperatures. Analysis of mitigation scenarios can inform national and international climate mitigation goals. For example, India’s National Action Plan on Climate Change includes recommendations for regulating the brick kiln sector [19]. The results of this present study underscore the importance of understanding and incorporating climate impacts in policies that call for the replacement of poor combustion kiln processes with cleaner kiln technologies. Further, this research suggests that regional impacts, such as those in the Arctic, should be considered when determining which kiln technologies are cleaner.

Using existing brick kiln CAA emissions data [1, 4] and temperature response factors [20], we estimate Arctic warming reductions from potential policy mitigation scenarios in South Asia. These scenarios involve strategies for replacing traditional brick kiln technologies with improved and cleaner burning kilns. Understanding how reductions in brick kiln emissions impact near-term climate can inform policy decision-making in India and other South Asian countries related to reducing risks to Arctic ecosystems and improving public health.

## 2. Methods

### 2.1. CAA EFs per kiln Type

A 2011–2012 field monitoring study [1, 4] directly measured emission factors (EFs) for elemental carbon (EC; a proxy for BC), OC, SO_2_, CO, and carbon dioxide (CO_2_) for 13 prominently used South Asian brick kilns. The current study focuses on CAA emissions, as the primary objective is to quantify the near-term Arctic temperature responses. Each of the 13 kilns was classified as either BTK, Zig-zag, VSBK, or DDK (an improved version of a clamp kiln). Given these EF measurements, the total annual brick production estimates [2], and the average brick weight produced in South Asia (2.9 kg per brick) [1, 4], they estimated the net South Asian emission masses per kiln type for the year 2012.

Since the previous study did not explicitly provide all the production-based EFs per kiln type, we calculated the average EF (*EF*_*k,i*_) per kiln type (*k* = BTK, Zig-zag, VSBK, and DDK) and pollutant (*i* = BC, OC, SO_2_, CO, and CO_2_) based on the total annual brick production estimates per kiln (*B*_*k*_) [2], the average brick weight (*W*) produced in South Asia (2.9 kg per brick) [1, 4], and the 2012 South Asian emission masses per kiln type and pollutant (*E*_*k,i*_) [1, 4] via the following equation:

(1) $$EF_{k,i}=\frac{E_{k,i}}{B_{k}\times W}.$$

These EF estimates are based on energy input and brick production and are reported in units of g kg^−1^ of fired brick. Supplemental table S8 of Weyant *et al* (2014) [1] provides the *E*_*k,i*_ and *B*_*k*_ used in equation 1. Briefly, of the estimated 260 billion clay bricks produced in South Asia in 2012, BTKs were responsible for the majority (70%), followed by traditional batch kilns (25%; approximated herein by DDK measurements), Zig-zag (3%), and VSBK (2%) [1]. Following a similar pattern, BTKs emitted the majority (~87%) of the total annual CAA mass emissions, followed by DDK (10%), VSBK (2%), and Zig-zag (1%) [1].

### 2.2. Estimating near-term Arctic temperature impacts from CAA emissions

To estimate the CAA’s impact on Arctic (defined here as latitudes between 60–90 °N) surface temperatures, we utilized six previously derived Arctic radiative forcing effects based on CAA emissions from seven regions and six source sectors [20]. Briefly, these relationships between emissions and temperatures north of 60° were derived from four different climate models, calibrated to a global climate sensitivity of 2.9 °C. The region and sector of interest utilized for this analysis are East+South Asia and Energy+Industry+Waste (includes brick kilns), respectively. In a similar approach to Seay *et al* (2020) [21], we assumed linearity between emission mass inputs and climate response outputs, and derived Arctic equilibrium temperature response ratios (i.e., the Arctic temperature response per unit mass emission per year). The annual net Arctic temperature response (*ΔT*) per kiln type (*k* = BTK, Zig-zag, VSBK, and DDK) was then calculated by:

(2) $$\Delta T_{k}=\overset{6}{\underset{i=1}{\sum }}E_{k,i}\times R_{i},$$

where *E* denotes a given kiln’s annual South Asian emission mass [1, 4] and *R* denotes the Arctic equilibrium temperature response ratios for each of the six short-term radiative forcing effects (*i* = BC direct atmospheric radiative forcing, BC indirect forcing from snow and ice, direct forcing from OC and SO_2_, and indirect forcing from OC and SO_2_). These ratios were derived from 2010 sector emission and temperature responses from Sand *et al* 2015 [20]. Direct forcing from the CAAs refers to the warming/cooling from absorption/reflection of solar radiation in the atmosphere. BC indirect forcing refers to the reduction in albedo and increase in surface absorption of solar radiation from deposition on snow and ice, whereas the OC and SO_2_ indirect forcing refers to its impact on cloud lifetime and formation.

### 2.3. Estimating mass emissions and Arctic temperatures from brick kiln replacement scenarios

Using the 2012 kiln data as a baseline, we investigate changes in mass emission and Arctic temperatures based on two brick kiln replacement options. We consider replacements in which bricks produced by BTKs were instead produced by Zig-zag kilns, and bricks produced by DDK were instead produced by VSBKs. Both options replace an older and less technologically advanced kiln (BTK and DDK) with a newer and more advanced kiln (Zig-zag and VSKB). Both replacement options were previously proposed as realistic real-world mitigation approaches based on similarities in brick production scales and straightforward integration and conversion of kiln types in the region [1, 4]. A BTK to Zig-zag conversion scenario is especially promising given its low capital investments, easy integration with existing production process, and that BTKs can easily be retrofitted into Zig-zags given the similarities in kiln structure [4].

The hypothetical mass emission (*E*) per pollutant (*i* = BC, OC, SO_2_, CO, and CO_2_) for both brick kiln conversions was estimated by:

(3) $$E_{i}=B_{j}\times M\times W\times EF_{k}+B_{j}\times (1-M)\times W\times EF_{j},$$

where *B* is the number of bricks produced in 2012 by the older kiln types (*j* = BTK, DDK), *M* is the fraction of older kilns replaced with advanced kilns, *W* is the average brick weight in South Asia (2.9 kg per brick) [1, 4], and *EF* is the production-based emission factor in grams of pollutant per kilogram of fired brick for both the technologically advanced kilns (*k* = Zig-zag, VSKB) and older kilns (*j* = BTK, DDK). We consider two mitigation scenarios. The first, herein abbreviated Mit_est_, derives *M* based on the estimated market penetration of VSBK and Zig-Zag technologies in South Asia since 2012 based on recent legislation and regulations. We estimate the value of *M* based on recently reported conversion estimates within each of the four South Asian countries. The second scenario, herein abbreviated Mit_max_, sets *M* = 1 for both replacement options, which assumes a 100% conversion to the improved kiln technologies.

Given the hypothetical mass emission results from equation 3, updated temperature responses from BC, OC, and SO_2_ emissions were derived as described in equation 2. We then compared the emission and Arctic temperature response results from the 2012 baseline to those given the two mitigation scenarios. The Mit_est_ scenario demonstrates the ramifications of the penetration of improved kiln technologies since 2012 on air quality and Arctic temperatures, whereas Mit_max_ illustrates the potential for additional future mitigation.

## 3. Results

### 3.1. Kiln type efficiency: emission and temperature impact per brick production

The production-based EFs derived via equation 1 are listed in table 1. The traditional DDK has the largest BC and OC EFs and smallest SO_2_ EF, due to low energy efficiency of brick production (leading to high BC and OC emissions per brick produced) and the use of wood for fuel (leading to near-zero sulfur emissions). Compared against DDK, the BC and OC EFs for VSBK are smaller, whereas the SO_2_ EF is larger, meaning it is expected this mitigation scenario will realize a decrease in BC and OC emissions and an increase in SO_2_. For the BTK to Zig-zag conversion scenario, however, a decrease in all three CAAs is expected given BTK’s EFs are larger for each CAA. For the gaseous pollutants, a decrease in CO and CO_2_ emissions is expected for both conversion scenarios.

The six forcer response ratios (*R* variable in equation 2) derived for BC, OC, and SO_2_ from East+South Asia’s 2010 mass emissions and its corresponding Arctic temperature responses [20] from the Energy+Industrial+Waste sector are listed in table 2. Positive ratios indicate a warming influence, whereas negative ratios indicate Arctic cooling. The largest magnitude ratios are for the two positive BC forcers, each being an order of magnitude greater than either of the negative OC or SO_2_ forcers. In other words, the BC warming effect is much greater than the cooling effects from either OC or SO_2,_ per unit of emission. The BC direct response ratio has a larger warming influence per unit emission as compared to BC snow/ice, whereas the two indirect ratios from OC and SO_2_ have a larger cooling impact than their direct response ratio counterpart.

**Figure 1.**
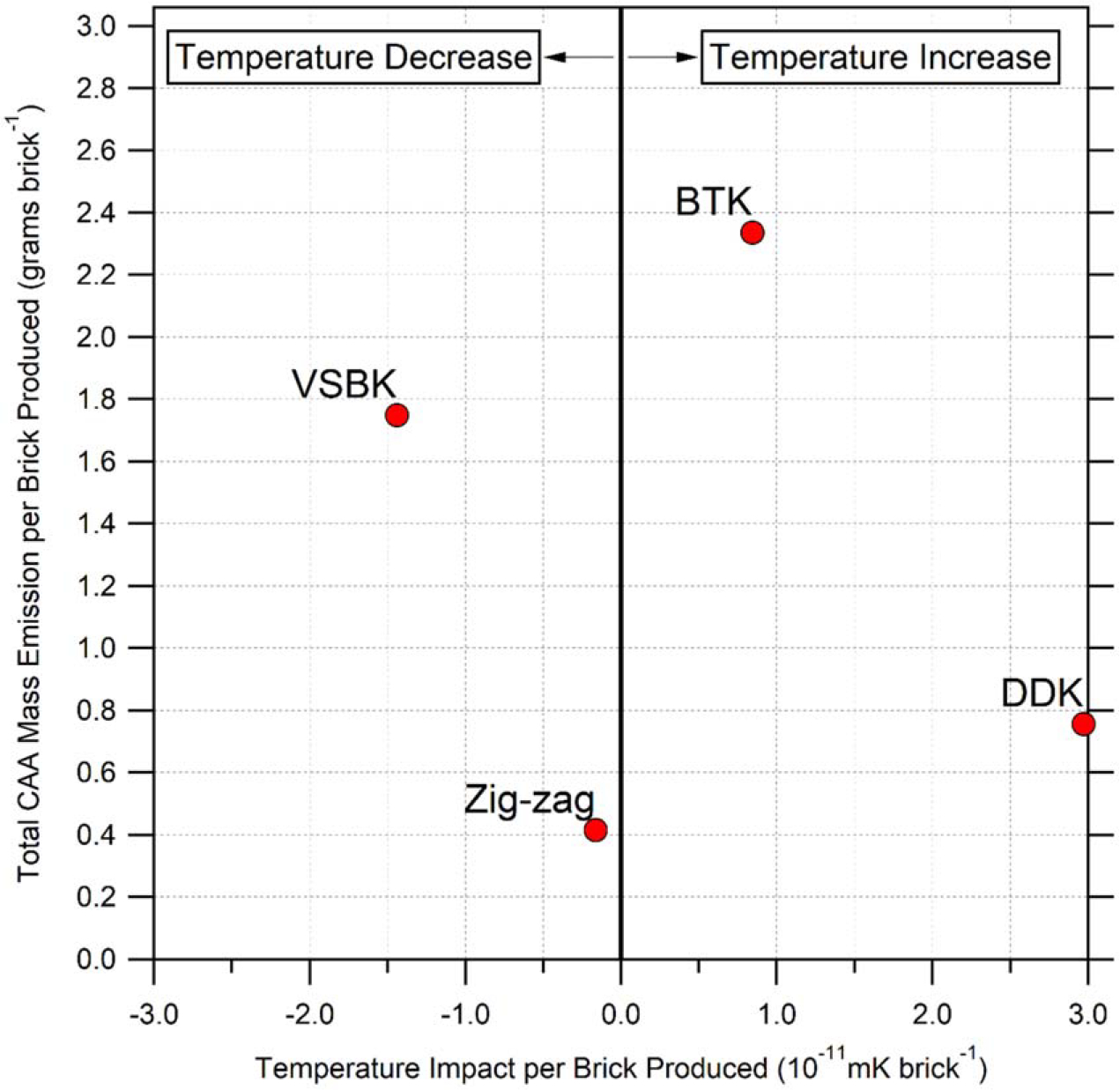
Per kiln CAA emission and near-term temperature impact. Net CAA (BC + OC + SO_2_) emissions per brick produced (y-axis) versus CAA Arctic temperature impact per brick produced (x-axis). The thick black vertical line illustrates no temperature impact, whereas markers to the left indicate temperature cooling and markers to the right indicate temperature warming. BTK = bull’s trench kiln; DDK = down draught kiln; VSBK = vertical shaft brick kiln.

Provided the production-based EFs, response ratios, and average weight of a South Asian brick, figure 1 illustrates the CAA mass emission and temperature impact per brick produced for each of the kiln types. Of the four kiln types, BTK is the dirtiest, emitting 2.3 grams of CAA per brick (g CAA/brick) produced. After BTK, the next dirtiest kiln is VSBK (1.7 g CAA/brick), followed by DDK (0.8 g CAA/brick), and Zig-zag (0.4 g CAA/brick). In terms of near-term temperature impact, both DDK and BTK have a net warming influence, whereas VSBK and Zig-zag both have a cooling influence. Zig-zags are the least environmentally impactful kiln, emitting less CAAs and influencing the Arctic climate less per unit brick produced than the other kilns. Figure 1 only considers influence from CAA emissions and does not account for air quality impacts from CO or long-term Arctic temperature responses from CO_2_.

### 3.2. Arctic temperature responses from baseline CAA emissions

**Figure 2.**
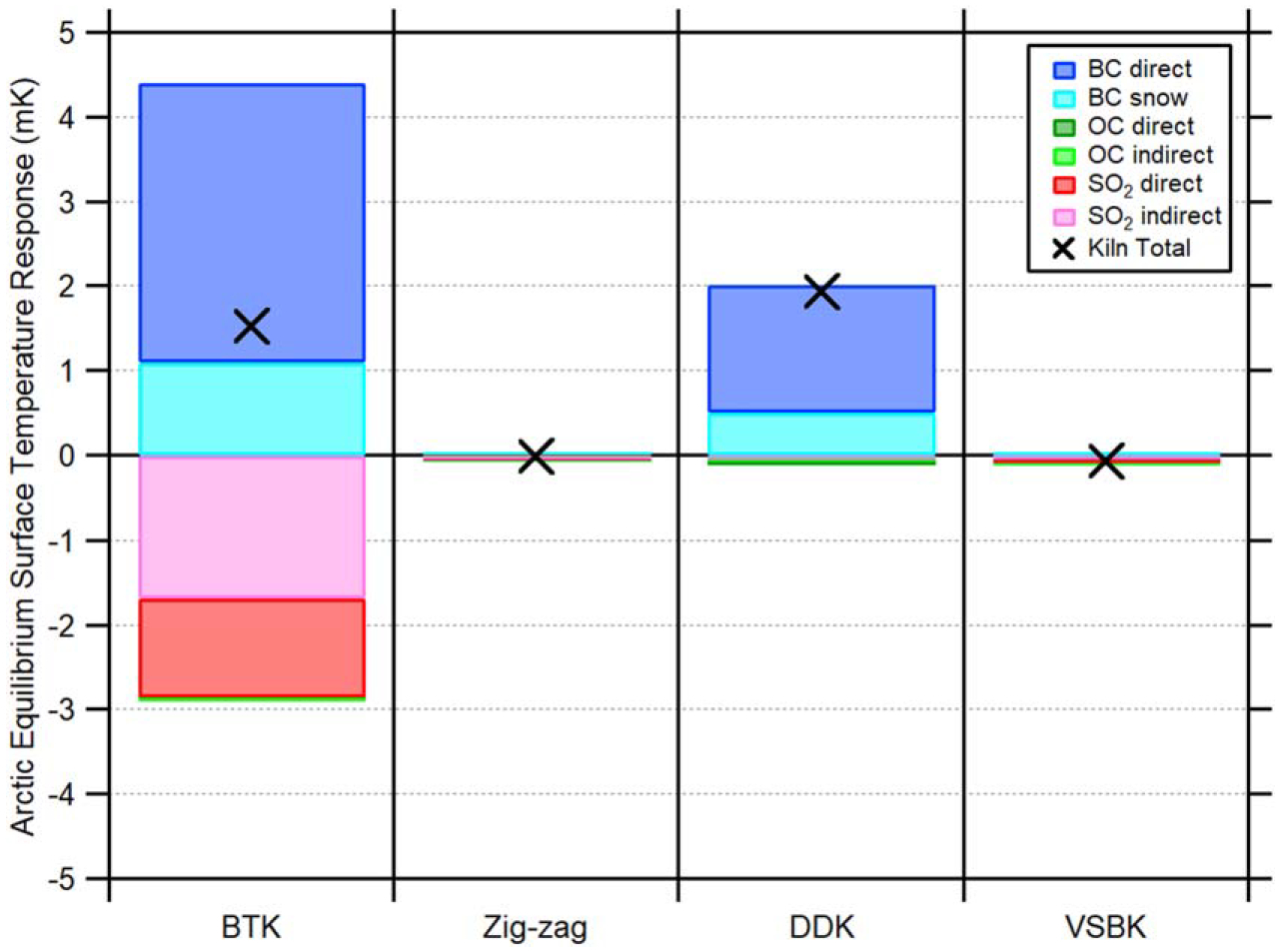
Model mean annual Arctic equilibrium surface temperature responses from various South Asian brick kiln CAA emissions. The six temperature forcers include BC direct (dark blue), BC snow/ice (light blue), OC direct (dark green), OC indirect (light green), SO_2_ direct (dark red), and SO_2_ indirect (pink). Positive y-axis values indicate a warming influence (BC forcers), and negative values indicate a cooling influence (OC and SO_2_ forcers). The black crosses represent the total temperature response for a given kiln type as derived by equation 2. The four kiln types, as represented from left to right on the x-axis, are bull’s trench kilns (BTKs), Zig-zag kilns, down draught kilns (DDKs), and vertical shaft brick kilns (VSBKs).

Figure 2 shows the annual mean Arctic surface temperature responses from the four brick kiln’s CAA emissions color-coded by the six temperature forcers. CAA emissions from BTK and DDK increased Arctic surface temperatures by +1.52 mK and +1.93 mK, respectively, while emissions from Zig-zag and VSBK resulted in slight cooling of −0.01 mK and −0.08 mK, respectively (black crosses on figure 2). While net baseline CAA emissions were more than eight times greater from BTK (420.55 Gg) as compared to DDK (49.15 Gg) kilns [1], the BC:(OC + SO_2_) ratio is such that the net warming impact is greater from DDK. The majority of BTK’s cooling aerosol emissions were SO_2_, which is reflected in the large negative temperature response (−2.86 mK) from the SO_2_ forcers (red and pink bars; figure 2).

Totaled across the four kilns, the 115 Gg of BC emissions [1] were responsible for +6.44 mK Arctic surface warming, whereas the 350 Gg of SO_2_ and 17 Gg of OC emissions resulted in −2.96 mK and −0.12 mK cooling, respectively. This resulted in an annual net warming of +3.36 mK to the Arctic surface when considering annual BC, OC, and SO_2_ emissions from all kiln types. The reduced BC emissions and cooling effects of OC and SO_2_ from Zig-zag and VSBKs were not large enough to offset the overall warming impact of the total brick kiln emissions. However, these results illustrate the near-term Arctic temperature mitigation potential of kiln replacement programs.

### 3.3. Brick kiln replacement scenarios: updated emissions and Arctic temperatures

To estimate the value of *M* in equation 3 for Mit_est_, we reviewed recently reported kiln conversion estimates within South Asia. In 2017, India’s Central Pollution Control Board issued a notification [22] requesting a shift to more efficient kiln types. The order estimated that roughly 16% (4,089 of 25,386) of kilns in Haryana, Punjab, Uttar Pradesh, and Rajasthan’s National Capital Region have been converted to Zig-Zag technology to date. In Bangladesh, from 2009 to 2017 the number of BTKs decreased from 4,500 to 2,373, a 47.3% reduction [23]. During this same time, Zig-Zags increased from 150 to 4,247. Anecdotal data indicates that in Pakistan, the Punjab province reported that 3,203 of the 8,554 brick kilns (37.4%) were converted to Zig-zags [24]. In Nepal, a 2015 earthquake damaged many of the country’s kilns, providing an opportunity to improve the kiln technology during reconstruction. Since then, it has been reported that approximately 10% of Nepal’s 1000 + kilns [25] have been converted to cleaner burning technologies [26].

It has been estimated that India, Bangladesh, Pakistan, and Nepal each contribute 81%, 9%, 9%, and 1%, respectively, to South Asia’s annual brick production [27]. Weighing each country’s estimated conversion percentages by its fractional contribution to annual brick production, we roughly estimate that since 2012 approximately 21% (((16*81) + (47.3*9) + (37.4*9) + (10*1))/100 = 20.68%) of older kiln technologies have been converted to the newer technologies in South Asia. In the Mit_est_ scenario, *M* is therefore set to 0.21 in equation 3 for both kiln type conversions. While the reported conversion percentages detailed here primarily focus on BTK to Zig-zag conversions, *M* = 0.21 is also used in the DDK to VSBK conversion given the lack of other quantitatively reported data. This fraction is likely biased high, as reports do indicate a slower adoption to using VSBK given its lower quality of brick and higher cost of conversion [25].

**Figure 3.**
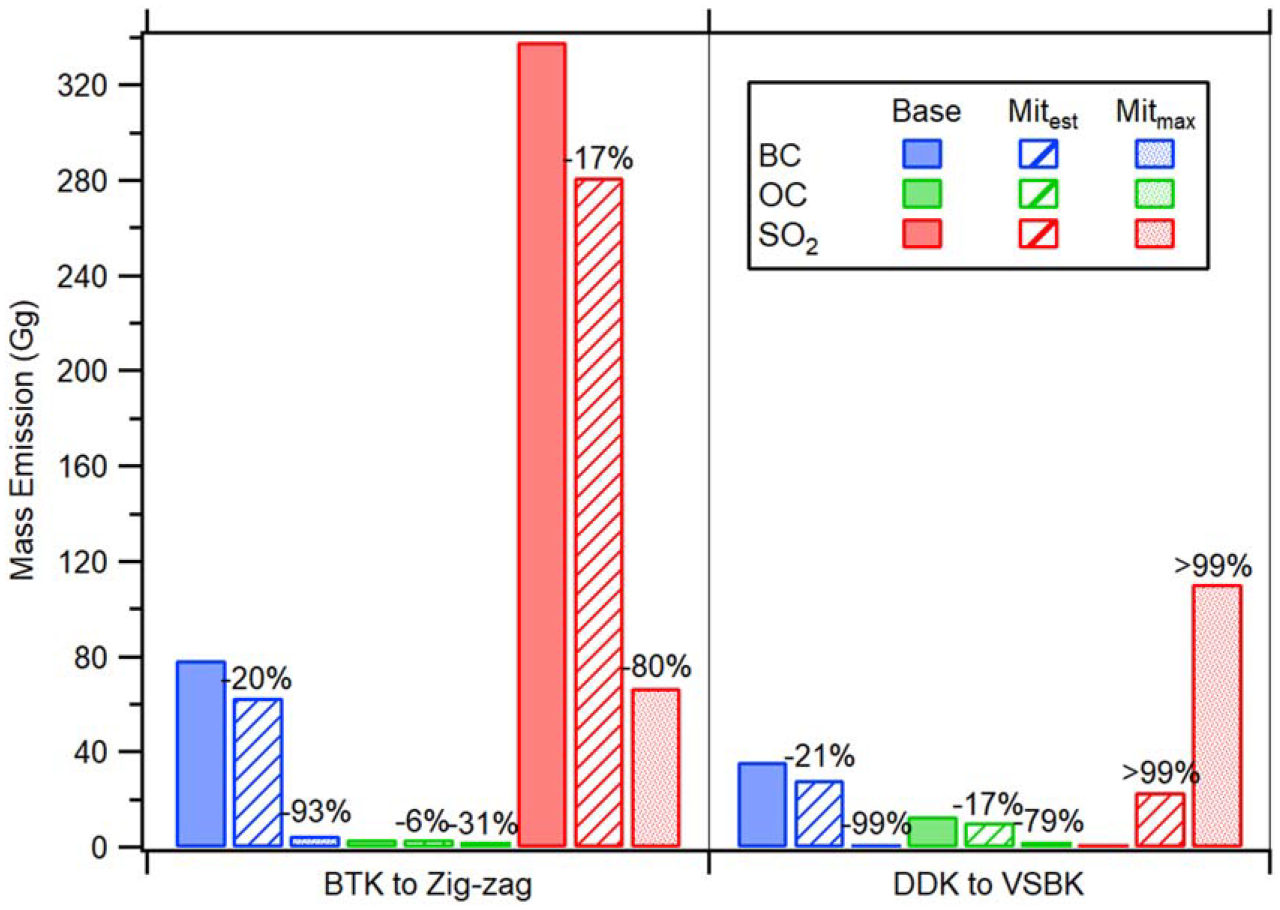
Mass emission comparison of BC (blue), OC (green), and SO_2_ (red) between the 2012 baseline (solid bars), Mit_est_ (striped bars), and Mit_max_ (spotted bars) assuming BTK conversions to Zig-zag (left) and DDK conversions to VSBK (right). Values above Mit_est_ and Mit_max_ bars indicate the scenario’s percent difference from the baseline.

Figure 3 compares CAA emissions between the 2012 baseline, Mit_est,_ and Mit_max_. For Mit_est_, the BTK to Zig-zag conversion realizes a decrease in emissions for each of the three CAAs, with a net decrease of 17%. If in the future South Asia replaces all BTKs with Zig-zags, Mit_max_ estimates the total net emissions can be reduced by 82% from the baseline. For the DDK to VSBK conversion, while Mit_est_ reduces both BC (21%) and OC (17%) emissions, this greatly increases emissions of SO_2_ (>99%), leading to a net increase in annual CAA emissions (28%). Net emissions would realize further increases if all DDKs were converted to VSBKs, as Mit_max_ estimates a 131% increase from the baseline. Considering both kiln replacements together, however, the total BC, OC, and SO_2_ emissions are reduced from the baseline and correspond to a 20%, 14%, and 10% reduction in emissions for the Mit_est_ scenario, respectively. This accounts for a 59.1 Gg (13%) reduction in net annual CAA emissions. For Mit_max_, total BC, OC, and SO_2_ emission reductions are much greater at 95%, 69%, and 48%, respectively, accounting for a net annual CAA decrease of 281.2 Gg (60%) from the baseline.

Compared to CAAs and particle phase emissions, total CO and CO_2_ emissions (not presented in figure 3) in the Mit_est_ scenario decreased from the baseline by 16% and 9%, respectively. From the BTK to Zig-zag conversion, a 12% CO decrease and 4% CO_2_ decrease was estimated, while the DDK to VSBK conversion resulted in an 18% decrease in CO and 16% decrease in CO_2_. Considering Mit_max_, total CO emissions could realize a 75% decrease from the baseline, whereas CO_2_ could decrease by 42%.

**Figure 4.**
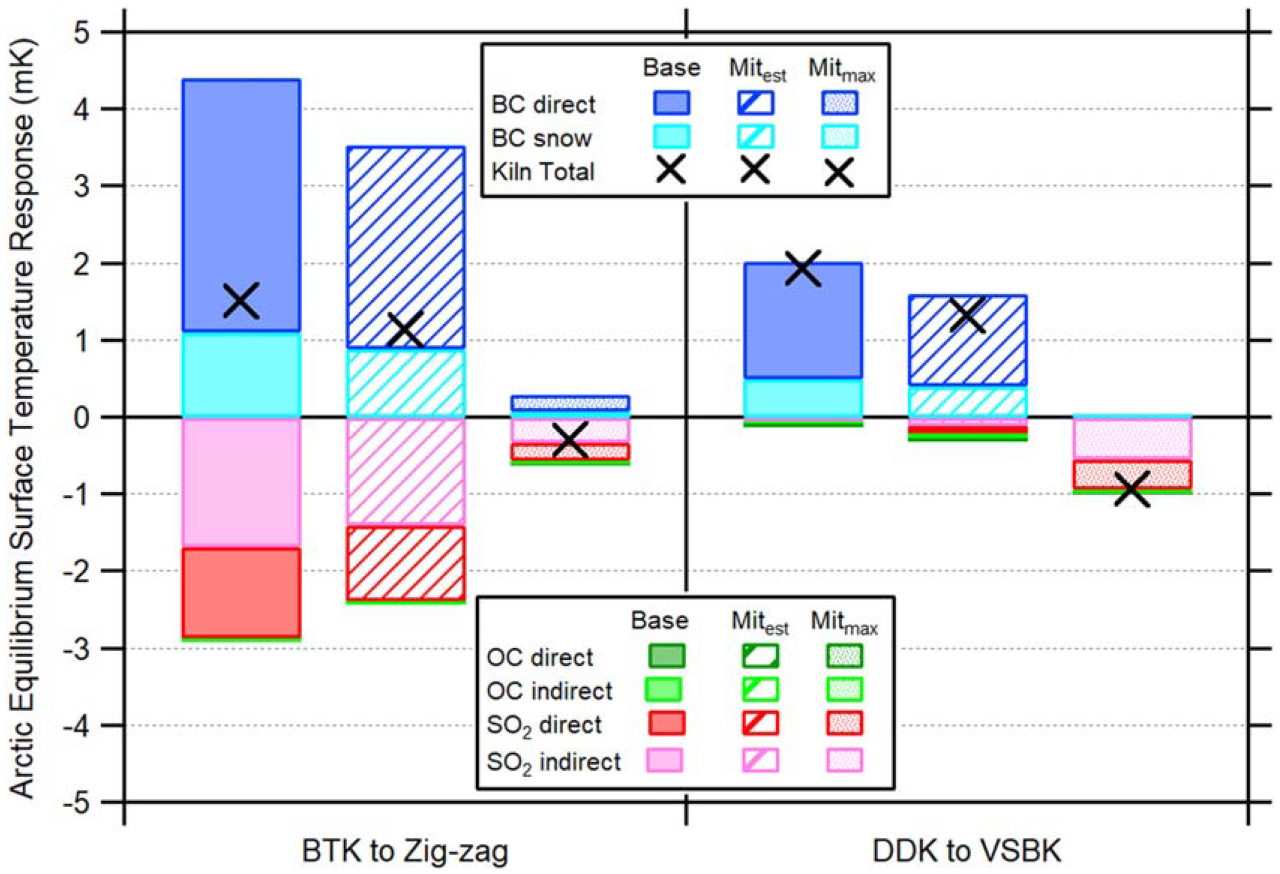
Annual Arctic equilibrium surface temperature response (units in mK) comparison of the six radiative forcing effects (BC direct [dark blue], BC snow/ice [light blue], OC direct [dark green], OC indirect [light green], SO_2_ direct [dark red], and SO_2_ indirect [pink]) between the 2012 baseline (solid bars), Mit_est_ (striped bars), and Mit_max_ (spotted bars) assuming BTK conversions to Zig-zag (left) and DDK conversions to VSBKs (right). Black crosses represent the sum of the six radiative forcing effects, i.e., the total Arctic surface temperature response per each scenario. Negative values indicate Arctic cooling given the pollutant’s forcing effect, whereas positive values indicate Arctic warming.

Estimated Arctic temperature response comparisons between the 2012 baseline, Mit_est_, and Mit_max_ are depicted in figure 4. Emission reductions for all three CAAs given the BTK to Zig-zag conversion (figure 3) results in a reduction in warming for the two BC forcers and a reduction in cooling for the four OC and SO_2_ forcers. Given the larger magnitude temperature impact from the BC forcers, the net warming response for a BTK to Zig-zag conversion (black cross [left], figure 4) in the Mit_est_ scenario is reduced by 25% from the baseline. However, this still results in an Arctic warming impact (+1.14 mK) compared to net zero emissions. If all of South Asia’s BTKs converted to Zig-zag (Mit_max_), however, temperatures are reduced by 119% and lead to −0.29 mK of cooling, helping combat the region’s overall anthropogenic temperature increase.

Both kiln type conversions have comparable reductions in BC and OC emissions, leading to a similar near-term Arctic temperature influence from these pollutants. However, the BTK to Zig-zag conversion realizes a large reduction in SO_2_, whereas DDK to VSBK leads to a large increase. Given SO_2_ is a cooling aerosol agent, the DDK to VSBK conversion’s increase in total SO_2_ emissions further enhances this conversion’s Arctic cooling impact. Therefore, emission changes for BC (reduced) and SO_2_ (increased) both result in a reduction in warming, whereas the decreased emissions of OC result in slightly enhanced warming. For the DDK to VSBK conversion, the net temperature response in the Mit_est_ scenario reduces warming by 31% from the 2012 baseline. For Mit_max_, warming is reduced by 149% from the baseline. The 2012 DDK emissions resulted in 1.93 mK of warming, whereas the conversion to VSBK in Mit_est_ reduces the warming to 1.33 mK and conversions in Mit_max_ would cool the Arctic by −0.94 mK.

Both kiln type conversions reduce the baseline’s Arctic warming effect. As opposed to the current warming of 3.45 mK from baseline emissions of BTK and DDK, when considering both kiln conversions, under Mit_est_ and Mit_max_ the Arctic would experience +2.46 mK of warming (a 29% reduction) and −1.23 mK of cooling (a 136% reduction), respectively. If also considering the baseline emissions from the Zig-zag and VSBK kilns along with the two conversions, the total Arctic cooling potential under Mit_max_ is −1.32 mK while producing the same number of bricks annually. While these reductions resolve only a small fraction of the estimated 1 K post-industrial anthropogenic warming [28], these results are meaningful considering this only investigates a single sector within a single region. To put these reductions in perspective, the 2012 US vehicle rule [29], enacted to reduce GHG emissions and increase mileage standards for one of the biggest global passenger car fleets, is expected to yield a temperature decrease of 12 mK by the end of the century [30].

With the expectation that market shares of clay-fired bricks will continue a similar trend in South Asia through 2030 [4] and building construction is to grow 6.6% annually through that same time [2, 4], in a business-as-usual (BAU) scenario it is estimated that brick production will more than triple from the 2012 baseline (2.6 *×* 10^11^ bricks produced) to 2030 (8.15 *×* 10^11^ bricks produced). Assuming this BAU scenario, the 2021 net CAA emissions are 857 Gg (a 78% increase from 2012), increasing the Arctic warming influence from 3.36 mK in 2012 to 5.97 mK in 2021. However, we estimate that to date approximately 21% of older South Asian kiln technologies have been converted to newer technologies. Given this shift in kiln type market shares and the 6.6% annual growth in brick production, updated 2021 emissions of CAA are 726 Gg (a 51% increase from 2012), which warms the Arctic by 5.38 mK (a 60% increase from 2012). This shows that the market penetration of advanced kilns, due in part to regulations implemented in South Asian countries, has mitigated 127 Gg of CAA emissions and 0.59 mK of Arctic warming in 2021 as compared to BAU. However, this has not been enough to keep up with the growing brick production demand, meaning net CAA emissions and Arctic temperatures still increased between 2012 and 2021. Looking beyond 2021, if all remaining BTKs and DDKs are replaced by 2030, annual CAA emissions would only increase 32% from the 2012 baseline as opposed to a 216% increase given BAU. Arctic temperatures would realize a cooling influence of −4.2 mK (relative to zero emissions), as compared to +10.6 mK of warming (a 14.8 mK difference) provided the BAU scenario.

## 4. Discussion

The results herein present timely and new information on the potential impacts of mitigation policies for brick kiln industries in South Asia. To our knowledge, prior studies have traditionally focused on financial performance, brick quality, air quality, environmental, or human health and safety impacts of brick kilns. This is the first study to investigate brick kiln emission’s impact on Arctic temperatures, a region more sensitive to climate change as compared to elsewhere [15–18]. The results suggest conversions in kiln technologies for this sector can reduce Arctic temperature in line with other specific sector’s emission reduction efforts. The climate analysis investigated BC direct atmospheric radiative forcing, BC indirect forcing from snow and ice, direct forcing from OC and SO_2_, and indirect forcing from OC and SO_2_. The study’s focus on non-CO_2_ radiative forcing pollutants illustrates the importance, role, and contribution of CAAs in the global climate system. Further, although the CAA emissions described are short-lived and from South Asia, its climate impacts in the Arctic illustrate the teleconnection and global links of the Earth’s climate system. When considering brick kiln emission mitigation options, regional and/or local policy action should consider global climate impacts along with local air quality and these other factors. To that end, a more nuanced understanding of brick kiln impacts is needed for more effective policy- or decision-making efforts.

Previous literature has discussed a DDK to VSBK conversion and this study shows that this scenario would help combat current anthropogenic Arctic warming. However, the practicality of this mitigation option from a policy perspective is more complicated. This conversion would realize an increase in CAA emissions; however, the decrease in the ozone precursor CO creates ambiguity in its net air quality impact. Also, the CAA emissions increase is solely from the large increase in SO_2_ (given DDK’s primary fuel source is biomass and VSBK’s is coal), as both BC and OC decrease in this scenario. While SO_2_ emissions are a public health concern for workers and surrounding communities [31] and have been associated with many of the same adverse health effects as PM, few epidemiological studies adequately differentiate the effects between these pollutants, making it difficult to compare on a per mass basis the health impacts between CAAs. Further, structural conversion from a DDK to VSBK is not straightforward, requires a larger monetary expense for kiln owners, and requires a change in primary fuel source. Combining this with a noted reduction in brick quality from DDK to VSBK [4], the incentives for end-users to make this kiln conversion are limited and recent reports [25]indicate a slow adoption to VSBK kilns. While the governments in India, Bangladesh, and Pakistan have issued some kiln conversion and emission standard requirements [22, 23, 32, 33], these generally focus on BTK to Zig-zag, and further government regulations or incentive would likely be needed to achieve DDK to VSBK conversions. The BTK to Zig-zag conversion offers a more realistic and feasible option, as it leads to improvements to both Arctic climate and local air quality (CAA and CO emission reductions), while also offering benefits as a business decision in that retrofitting BTKs to Zig-zags reduces conversion costs, brick quality is not sacrificed, and an overall greater return on investment can be expected [4]. Given the expected future growth in brick production, setting and meeting mitigation program goals are needed to substantially limit negative air quality and climate impacts.

## 5. Conclusions

For regions such as South Asia, where building construction, and therefore brick production, are expected to nearly double from 2020 to 2030 [2, 4], mitigation strategies are critical to prevent further impacts to air quality and Arctic climate change and achieve the co-benefits associated with improved air quality, health, and climate goals. Using previously derived CAA emissions [4] from four South Asian brick kilns (BTK, Zig-zag, VSBK, and DDK), we estimated Arctic temperature impacts from these baseline emissions and Arctic temperature and mass emission mitigation potentials considering two kiln conversions in two mitigation scenarios (Mit_est_ and Mit_max_). Baseline CAA emissions accounted for an annual near-term Arctic warming of 3.36 mK, in which BTK and DDK provided a large warming influence and Zig-zag and VSBK a small cooling influence.

We estimate the current BTK to Zig-zag conversions under the Mit_est_ scenario have realized an annual reduction of 72.6 Gg of CAA, whereas a 100% conversion (Mit_max_) could realize a 345.7 Gg reduction. Current DDK to VSBK conversions have increased annual emissions by 13.5 Gg, given its large increase in SO_2_ output, while the Mit_max_ scenario would increase emissions by 64.5 Gg. Under Mit_est_, the CAA emission changes have reduced Arctic warming by 0.4 mK (BTK to Zig-zag) and 0.6 mK (DDK to VSBK), but both still increase Arctic temperatures (relative to net zero emissions). The potential CAA emission changes under Mit_max_, however, would reduce Arctic warming by 1.8 mK (BTK to Zig-zag) and 2.9 mK (DDK to VSBK), both of which result in an Arctic cooling influence (−0.29 mK and −0.94 mK, respectively). However, while a conversion from DDK to VSBK reduces Arctic warming, the air quality (in terms of CAAs) is reduced given the large increase in sulfate. Both factors must be considered when determining a mitigation solution. Both kiln conversion types realize large decreases in CO and CO_2_ emissions.

While baseline CAA emissions result in 3.36 mK of Arctic warming, given the kiln conversion progress to date, current CAA emissions are reduced to 360.3 Gg (12.3% reduction) and the Arctic warming influence is reduced to 2.4 mK (29.3% reduction) when considering the same annual brick production as the baseline. If all BTKs and DDKs are converted to Zig-zags and VSBKs, respectively, CAA emissions would reduce to 200.85 Gg (58% reduction) and cool the Arctic by −1.32 mK (139% reduction). However, when considering the annual 6.6% increase in the number of bricks produced, while the estimated 21% of kiln conversions to date help mitigate the increased production, it is not enough to fully offset, meaning emissions are larger and Arctic temperatures are warmer today than compared to the baseline year. If all kiln conversions are complete by 2030, however, while the net annual emissions will still be increasing, the Arctic will have realized a net cooling impact (relative to zero emissions).

These timely results provide insight into brick kiln mitigation strategies and their impacts on emissions and near-term Arctic temperature responses in South Asia. This work also demonstrates an analysis technique that can easily be implemented to assess emission and temperature influence from other sectors and regions around the world. While this study focuses on the near-term climate and air pollution impacts from CAAs, the substantial emissions of the long-term GHG CO_2_ and the hazardous gas CO must be further investigated to better understand these mitigation scenario impacts. Future studies could quantify the long-term Arctic temperature and human health impacts of these kiln conversion’s reductions in CO_2_ and CO emissions, respectively. This investigation would provide a more complete understanding of the impacts from these mitigation scenarios. In addition, further studies could also compare on-site worker exposures, other risks to safety, seasonal emissions, and production output differences between kiln types to further assess kiln conversion practicability. Lastly, this study only focuses on kiln replacement as a mitigation option. Other opportunities for emission and climate mitigation include the use of clean fuel alternatives (e.g., clean coal, natural gas, biofuels), kiln operator training and operating practice improvements, and the use of alternative building materials during kiln construction [34].

## Figures and Tables

**Table 1.** Production-based EFs (g kg^−1^ brick) per kiln type.^a^

Kiln Type	BC	OC	SO_2_	CO	SO_2_
BTK	0.1507	0.0068	0.6481	2.229	124.3
Zig-zag	0.0100	0.0048	0.1286	0.9619	103.1
DDK	0.1917	0.0691	1.6E-05	13.20^b^	271.1
VSBK	0.0015	0.0144	0.5870	2.187	69.13

3 EFs calculated from 2012 emission and brick production estimates derived by Weyant *et al* (2014) [1].

4 CO EF from DDK taken directly from table 2 of Weyant *et al* (2014).

**Table 2.** Model mean annual arctic surface temperature forcer response ratios (mK Gg^−1^) from South and East Asia emissions in the energy+industrial+waste sector.^a^

CAA	BC		OC		SO_2_	
Temperature Forcer	Direct	Snow/Ice	Direct	Indirect	Direct	Indirect
Response Ratio (mK Gg^−1^)	0.04200	0.01400	−0.00153	−0.00574	−0.00350	−0.00496

5 Ratios based on 2010 sector emissions and temperature responses derived by Sand *et al* (2015) [20].

## Data Availability

All data that support the findings of this study are included within the article (and any supplementary files).
